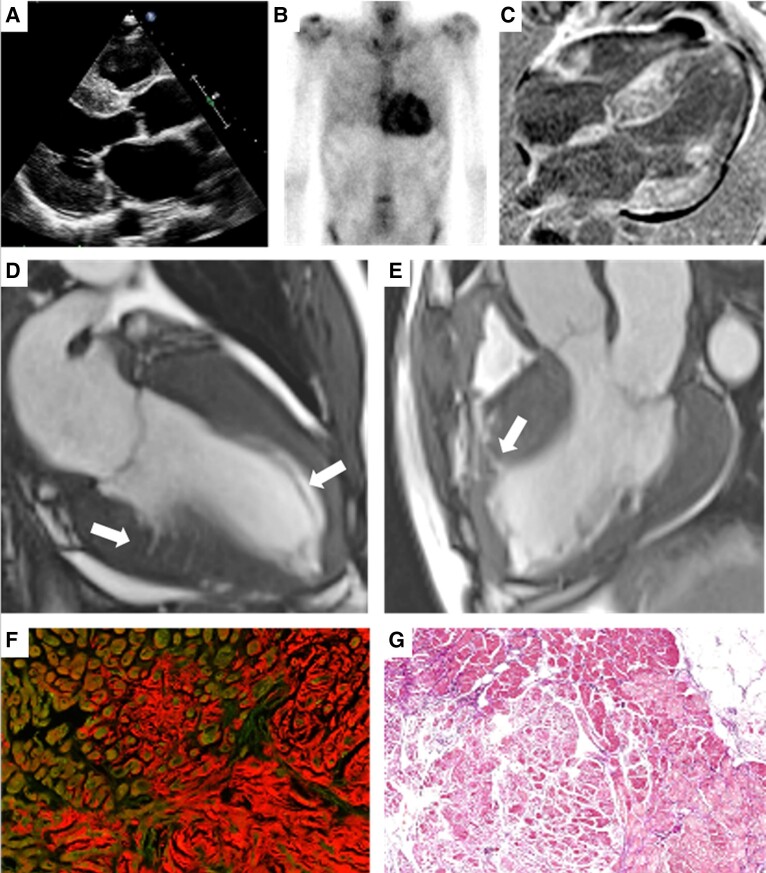# Beyond hypertrophy: unmasking sarcomeric hypertrophic cardiomyopathy in a patient with wild-type ATTR amyloidosis

**DOI:** 10.1093/ehjci/jeae321

**Published:** 2024-12-11

**Authors:** Anne J Koppelaar, Michelle Michels, Alexander Hirsch

**Affiliations:** Department of Cardiology, Thorax Center, Cardiovascular Institute, Erasmus Medical Center, Dr. Molewaterplein 40, 3015 GD Rotterdam, The Netherlands; Department of Radiology and Nuclear Medicine, Erasmus Medical Center, Dr. Molewaterplein 40, 3015 GD Rotterdam, The Netherlands; Department of Cardiology, Thorax Center, Cardiovascular Institute, Erasmus Medical Center, Dr. Molewaterplein 40, 3015 GD Rotterdam, The Netherlands; Department of Cardiology, Thorax Center, Cardiovascular Institute, Erasmus Medical Center, Dr. Molewaterplein 40, 3015 GD Rotterdam, The Netherlands; Department of Radiology and Nuclear Medicine, Erasmus Medical Center, Dr. Molewaterplein 40, 3015 GD Rotterdam, The Netherlands

A 72-year-old male presented with new-onset heart failure. Initial evaluation showed low-voltage complexes on the electrocardiogram and left ventricular hypertrophy on transthoracic echocardiography (*Panel A*). Given the suspicion of amyloidosis, bone scintigraphy was performed, revealing myocardial tracer uptake (Perugini Grade 3) (*Panel B*). Further testing excluded the presence of monoclonal protein conforming the diagnosis of transthyretin amyloidosis (ATTR).

Further phenotyping using cardiovascular magnetic resonance (CMR) imaging showed diffuse late gadolinium enhancement of the left and right ventricle and interatrial septum consistent with amyloidosis (*Panel C*). However, there were also hallmark features of sarcomeric hypertrophic cardiomyopathy (HCM), including reverse septal curvature, myocardial crypts, muscular band, and an irregular wall pattern (*Panels D and E*). Subsequently, DNA analysis was performed including the TTR gene but also the genes involving HCM and identified a pathogenic variant in the *MYBPC3* gene. No pathogenic variants were found in the TTR gene.

The myocardial biopsy revealed amyloid deposits (*Panel F*), alongside myocardial hypertrophy and fibrosis (*Panel G*), consistent with a MYBPC3 variant.

This case highlights a rare combination of wild-type ATTR amyloidosis and sarcomeric HCM due to a pathogenic variant in the MYBPC3 gene. It underscores the importance of comprehensive phenotyping of cardiomyopathies. CMR was essential in identifying features of both amyloidosis and sarcomeric HCM. The discovery of a pathogenic *MYBPC3* variant has significant familial implications, emphasizing the need for genetic counselling and cascade screening. CMR is indispensable in the work-up of left ventricular hypertrophy, not only in achieving diagnostic clarity but also in informing family-based risk evaluation and long-term management strategies.

**Funding:** None declared.

**Data availability:** No new data were generated or analysed in support of this research.

**Figure:**